# Does the Occurrence of Particular Symptoms and Outcomes of Arterial Ischemic Stroke Depend on Sex in Pediatric Patients?—A Pilot Study

**DOI:** 10.3390/brainsci10110881

**Published:** 2020-11-20

**Authors:** Ilona Kopyta, Anna Dobrucka-Głowacka, Agnieszka Cebula, Beata Sarecka-Hujar

**Affiliations:** 1 Department of Pediatric Neurology, Faculty of Medical Sciences in Katowice, Medical University of Silesia in Katowice, Medykow Str 16, 40-752 Katowice, Poland; ilonakopyta@autograf.pl; 2 Upper-Silesian Child’s Health Center, Medykow Str 16, 40-752 Katowice, Poland; aniadobrucka@yahoo.com (A.D.-G.); agnieszkaszew@wp.pl (A.C.); 3 Department of Basic Biomedical Science, Faculty of Pharmaceutical Sciences in Sosnowiec, Medical University of Silesia in Katowice, Kasztanowa Str 3, 41-200 Sosnowiec, Poland

**Keywords:** arterial ischemic stroke, children, sex, post-stroke, outcome, consequence, deficit

## Abstract

Arterial ischemic stroke (AIS) in childhood is reported to occur more frequently in boys, which may lead to the assumption that the prevalence of post-stroke deficits is sex related. The present study aimed to evaluate sex-related differences in functional outcomes (hemiparesis, seizures, aphasia, and motor disturbances other than hemiparesis) in pediatric patients with AIS. A total of 89 children (52 boys and 37 girls; mean age at stroke onset: 8.4 ± 5.6 years) were evaluated retrospectively based on data from medical records. The patients were divided into subgroups according to age (i.e., infants and toddlers, children, and adolescents), stroke subtype (i.e., lacunar anterior circulation infarct (LACI), total anterior circulation infarct (TACI), partial anterior circulation infarct (PACI), posterior circulation infarct (POCI)) and stroke location (i.e., anterior stroke, posterior stroke). Significant differences in the prevalence of stroke subtypes between girls and boys were observed (*p* = 0.034). POCI stroke were found to be more frequent in boys than in girls (OR = 8.57 95%CI 1.05–70.23, *p* = 0.023). Males predominated in the total group and in all analyzed age subgroups. The proportions of boys within the subgroups according to stroke subtype were extremely high for the POCI and TACI stroke subgroups. On the other hand, girls predominated in the LACI stroke subgroup. Frequency of central type facial nerve palsy and other symptoms of AIS were found to significantly differ between male subgroups according to stroke subtype (*p* = 0.050 and *p* < 0.001, respectively), as well as between children with anterior stroke and those with posterior stroke (*p* = 0.059 and *p* < 0.001, respectively). Post-stroke seizures appeared significantly more commonly in girls with TACI and POCI stroke than in girls with LACI and PACI stroke (*p* = 0.022). In turn, the prevalence of post-stroke hemiparesis differed between stroke subtypes in boys (*p* = 0.026). In conclusion, sex may have an impact in predisposing to a certain type of AIS in the patient. Post-stroke seizure may be related to stroke subtype in girls and hemiparesis in boys. However, further studies are needed to confirm the results.

## 1. Introduction

Although most cases of arterial ischemic stroke concern the adult population, it is not an unprecedented diagnosis among children [1,2], and is associated with a high mortality and the risk of long-term consequences. Childhood AIS affects about 1.2–2.4 per 100,000 children per year [3,4,5]. Mortality during the acute phase of childhood AIS has decreased, but cerebrovascular diseases are still among the top 10 causes of death in the pediatric population in the United States [6,7]. In-hospital death related to childhood AIS occurs in 2.6% patients [8]. The most common risk factors of AIS in children are congenital heart defects, infections, prothrombotic state [5,8], and focal cerebral arteriopathy of childhood (FCA), which may have an impact on post-stroke consequences [9].

AIS carries detrimental neurological consequences, which affect all aspects of patients’ lives, and entail the costs of medical care and rehabilitation. The most common consequences in the pediatric population are as follows: motor disabilities, speech impairment, epilepsy or seizures, and stroke recurrence [10,11].

The role of sex in post-stroke outcomes is as yet unclear, especially in the pediatric population. In adult patients, AIS outcomes are often more severe in women than in men with respect to motor disability [12], but the differences cannot be explained only by the level of circulating female hormones [13,14,15,16].

In studies concerning the childhood population, a higher prevalence of AIS in male subjects is usually described. However, an analysis of the current literature and an observation of the recruited group of AIS children led to the hypothesis that sex may affect both the frequency of symptoms in the acute phase of the disease and the neurological post-stroke deficits.

The aim of the present study was to evaluate possible sex-related differences in functional post-stroke outcomes (hemiparesis, seizures, aphasia, and motor disturbances other than hemiparesis) in a population of pediatric patients with AIS, regarding age and stroke subtype. In addition, AIS symptoms were also compared between girls and boys suffering from AIS.

## 2. Materials and Methods

### 2.1. Study Participants

We retrospectively analyzed 89 children with AIS hospitalized in the Department of Pediatric Neurology of the Medical University of Silesia in Katowice (Poland) between 2002 and 2013. The subjects were selected based on an initial search for International Classification of Diseases 10 (ICD-10) medical diagnosis groups of I63 and I64 in the department register of admissions and discharges, as well as the medical software used for managing patients’ hospitalization, followed by later confirming the set diagnosis and checking for inclusion/exclusion criteria in individual medical histories. As the study was retrospective, the clinical data were limited. The inclusion criteria were as follows: (a) diagnosis of AIS in the acute phase based on clinical and radiological criteria (computed tomography (CT) and/or magnetic resonance imaging (MRI) results confirming the diagnosis); (b) the age at the acute phase of stroke, ranging from 1 month to 18 years; and (c) available data on neurological post-stroke deficits. In turn, children were excluded in the case of (a) a lack of CT and/or MRI results confirming the diagnosis and (b) skull injury as a cause of AIS. In the analyzed group, there were no patients with sickle cell disease, moyamoya or mitochondrial myopathy, encephalopathy, lactic acidosis, or stroke-like episodes (MELAS) syndrome, because patients with these conditions are extremely rare in our geographical region; e.g., moyamoya was estimated to affect 0.089/100,000 individuals in Poland [17].

The study was conducted in accordance with the Declaration of Helsinki, and the Ethics Committee of the Medical University of Silesia in Katowice gave opinions on the study (KNW/0022/KB/88/18). Since the study has a retrospective character, no patients were recruited and thus no written informed consents were needed.

### 2.2. Neurological Examinations

For the purpose of this study, two results of neurological examination and neuroimaging were taken into consideration (at the acute phase and during the follow-up). At the department, the neurological examination of patients with pediatric AIS was performed and documented by either a specialist in pediatric neurology or a trainee in pediatric neurology under supervision, at least once a day at the acute phase of the disease. The authors adopted the results from admission as the baseline rating (which, in most cases, was also the day of the most severe symptoms). Another evaluation, after the acute phase of stroke, was performed at different time points for each patient. The authors decided to consider the latest available data as being the most representative for the AIS outcome. Similarly, in the case of neuroimaging, the first data are from patient admission in the acute phase, whereas the follow-up time points differed for each child. As, in some cases, neuroimaging was performed more than twice, the authors took into consideration the last results available.

The stroke subtypes were evaluated using the Oxfordshire classification [18] and included the following: LACI, TACI, PACI, POCI.

### 2.3. Criteria for Dividing Participants into Subgroups

The age of analyzed patients covers a wide range (i.e., from early infancy to early adulthood), which results mainly from the rarity of the disease. Therefore, we attempted to overcome the age problem by dividing patients according to age. In the analyzed group of patients, three age subgroups were recognized, which has a physiological basis, namely: (a) infants and toddlers (28 days old to 35 months old, mean age of 1.3 years ± 0.6; *n* = 17), (b) children (3–11 years old, mean age of 6.4 years ± 2.6; *n* = 41), and (c) adolescents (12–18 years old, mean age of 15 years ± 1.6; *n* = 31).

In addition, four subgroups in regard to stroke subtypes were classified, i.e., (a) LACI, (b) TACI, (c) PACI and (d) POCI. Lastly, the patients were also divided into two subgroups regarding the location of the stroke: (a) anterior stroke (AS) and (b) posterior stroke (PS).

### 2.4. Consequences of Ischemic Stroke

The median follow-up time in the whole group was 2 years (interquartile range (IQR) 4 years; 1–5 years). In the present study, the observed neurological consequences of AIS were divided into groups, as follows: hemiparesis (as most of the children suffered from AIS within the anterior part of brain vasculature, and hemiparesis was the most typical consequence), seizures, aphasia, and movement disorders other than hemiparesis. Next, each patient was evaluated for the number of occurring consequences (i.e., “none” and “one or more” categories). Because of the retrospective character of the study, the assessment for each of the above consequence took place at different follow-up time points for each patient. The definition of post-stroke seizures in pediatric patients with AIS was adopted from the study by Beslow et al. [19]. Seizures were recognized as early/acute symptomatic seizures (up to seven days after stroke onset), remote symptomatic seizures (beyond seven days from stroke presentation) and epilepsy, when at least two recurrent, non-provoked seizures occurred after the acute phase of stroke. The term “seizures” was used in relation to all patients who experienced any type of seizure, early or late.

All comparisons of the outcome prevalence were made between the whole sex subgroups and, additionally, in subgroups according to both particular age ranges and stroke subtype.

### 2.5. Statistical Analysis

STATISTICA 13.0 software (STATSOFT; Statistica, Tulsa, OK, USA) was used to perform the statistical tests. The mean values (M) and standard deviations (SD) were estimated for continuous variables, while the absolute numbers (n) and relative numbers (%) were estimated for the categorical variables. The U Mann–Whitney test was used to compare the continuous variables between the boys with AIS and the girls with AIS. The dichotomous categorical variables between the girls and boys were compared using the 2-tailed Fisher’s exact probability test. In turn, to analyze polytomous categorical variables between subgroups, the Freeman–Halton extension of the Fisher exact test was used (VassarStats). The value of *p* ≤ 0.05 was considered to be statistically significant. An odds ratio (OR) with 95% confidence interval (CI) was calculated for the occurrence of POCI stroke, prevalence of post-stroke seizures in girls with POCI and TACI stroke and prevalence of post-stroke hemiparesis in boys with TACI and PACI stroke. For the proportion of male subjects in the total group of patients as well as subgroups, upper and lower limits of 95%CI were assessed.

A power analysis was performed during the study, on the basis of differences in several parameters, i.e., birth weight, frequencies of stroke subtypes, prevalence of other symptoms of AIS as well as presence of seizures, aphasia and motor disturbances other than hemiparesis in the follow-up between boys and girls in age subgroups using a two tailed test with a significance level of 0.05. Different values of power for different parameters were obtained with a range between 65% and 80%. Thus, the power was sufficient based on this assumption. The sample size of 89 pediatric patients with AIS which we included in the study was satisfactory for us and similar group sizes are reported by other authors. In the case of rare diseases, which include childhood AIS, achieving a large sample size in one medical center is limited.

## 3. Results

### 3.1. Characteristics of the Study Group

In the present study, 52 out of 89 analyzed children with AIS were boys (i.e., 58%). The proportions of male subjects within the total group as well as the subgroups according to age and stroke subtype are demonstrated in Table 1.

We observed that male sex predominated in all analyzed age subgroups. However, the greatest proportion of male subjects was in the subgroup of the youngest patients, i.e., the infants–toddlers group and next the adolescents. In turn, the male proportion within subgroups according to stroke subtype was extremely high for the POCI and TACI stroke subgroups. On the other hand, girls predominated in the LACI stroke subgroup (Table 1).

Table 2 demonstrates the general characteristics of the examined group of patients. The differences in age at stroke onset and birth weight between girls and boys were not significant.

**Figure 1:**
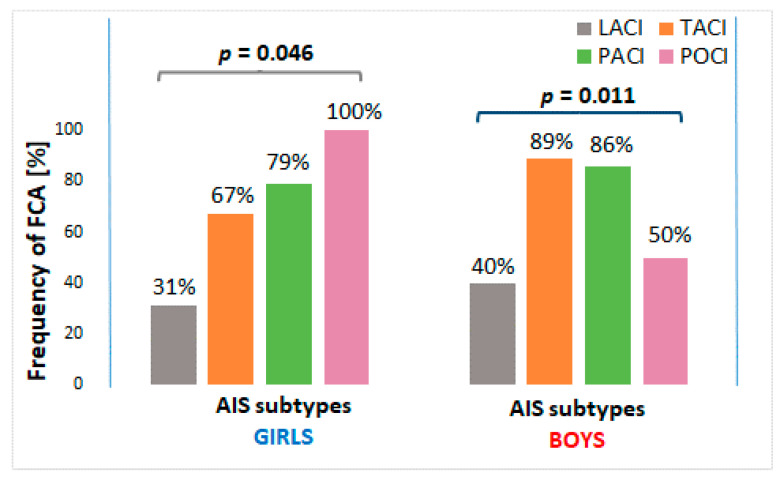
Frequency of FCA in girls and boys according to stroke subtypes. FCA—focal cerebral arteriopathy of childhood; AIS—arterial ischemic stroke; LACI—lacunar anterior circulation infarct; TACI—total anterior circulation infarct; PACI—partial anterior circulation infarct; POCI—posterior circulation infarct.

The frequency of heart disease was similar in girls and boys. FCA was observed in 71% of boys and in 60% of girls. However, the observed differences revealed no statistical significance. Furthermore, no significant differences in the prevalence of heart disease or FCA were found between girls and boys in subgroups according to age and stroke subtypes. However, in girls, the prevalence of FCA significantly differed between stroke subtypes (*p* = 0.046). FCA was present in 79% of girls with PACI stroke and 67% of girls with TACI stroke, while only in 31% of girls with LACI stroke. The only girl with POCI stroke also had FCA. Similarly, FCA was related to stroke subtype in boys (*p* = 0.011). In male patients, FCA was also more frequently present in cases with TACI and PACI stroke (89% and 86%, respectively), while in 50% of male cases with POCI stroke (Figure 1).

**Figure 2:**
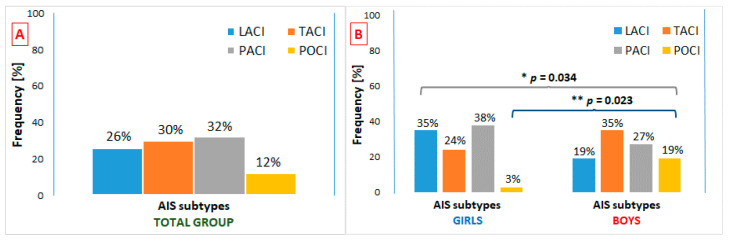
Frequencies of AIS subtypes in total group (**A**) and in sex subgroups (**B**). AIS—arterial ischemic stroke; LACI—lacunar anterior circulation infarct; TACI—total anterior circulation infarct; PACI—partial anterior circulation infarct; POCI—posterior circulation infarct; *—difference in overall distributions of stroke subtypes between girls and boys; **—difference in the prevalence of POCI stroke between girls and boys.

The prevalence of stroke subtypes was significantly different between girls and boys (*p* = 0.034). The most common type of stroke in AIS boys was TACI (18 boys out of 52 analyzed boys with AIS), whereas in the girls it was PACI (14 girls out of 37 AIS girls) (Figure 2). In addition, the prevalence of POCI stroke type significantly differentiated sex subgroups (*p* = 0.023), as it was present in ten boys and only in one girl. The odds for a boy to have POCI stroke were found to be over eight-fold higher than for a girl (OR = 8.57 95%CI 1.05–70.23, *p* = 0.023).

### 3.2. Analysis of the Prevalence of AIS Symptoms

In the total group, the most common symptoms at stroke onset both in girls and boys were hemiparesis, central type facial nerve palsy, consciousness disturbances, and aphasia. No significant differences in the distribution of particular AIS symptoms between girls and boys were found. Paresis as a symptom of AIS was present in all girls and in 81% of boys from the children group (from 3 to 11 years) (*p* = 0.051). Other AIS symptoms were more frequent in adolescent boys than in the girls from this age group (47% vs. 17), but again the difference did not reach a statistical significance. When we analyzed the distribution of AIS symptoms according to stroke subtype separately for girls and boys, we observed that central type facial nerve palsy and others than the analyzed AIS symptoms significantly differed between AIS subtypes in boys (*p* = 0.050 and *p* < 0.001, respectively). In turn, no significant differences in frequencies of stroke symptoms in regard to stroke subtype were demonstrated in girls (Table 3).

### 3.3. Analysis of the Prevalence of Post-Stroke Consequences

The median time the girls were followed up was 3 years (IQR 5 years; 1–6 years), whereas the median time of the follow-up period for boys was 2 years (IQR 2 years; 1–3 years). This difference was not significant. One girl with a congenital heart defect (common atrioventricular canal) died during the follow-up in the perioperative period after a hemi-Fontan procedure.

The post-stroke seizures and persistent hemiparesis occurred in 27% and 73% of girls, respectively, compared to 19% and 64% of boys, respectively. In turn, aphasia was observed in 14% of all boys and in 5% of all girls. However, the appearance of any of the post-stroke consequences among the studied patients did not differentiate girls from boys in the total group or in the subgroups with regard to age and stroke subtypes.

Differences in the prevalence of post-stroke outcomes according to stroke subtype analyzed separately in the girls and boys subgroups are shown in Table 4. We observed that seizures after stroke appeared significantly more commonly in girls with TACI stroke, while only in one girl with LACI stroke and only one girl with POCI stroke (*p* = 0.022). The odds of a girl with POCI or TACI stroke having post-stroke seizures was over eight-fold higher than for a girl with LACI or PACI (OR = 8.62 95%CI 1.65–44.99, *p* = 0.017). In turn, post-stroke hemiparesis differed between stroke subtypes in boys (*p* = 0.026). The highest prevalence of hemiparesis was observed in the case of boys with TACI and PACI stroke (89% and 64%, respectively) compared to boys with LACI and POCI stroke (OR = 5.36 95%CI 1.57–18.25, *p* = 0.008).

### 3.4. Neurological Symptoms and Consequences of AIS in Subgroups Due to AIS Location

The location of the ischemia may determine the symptoms during AIS onset, as well as post-stroke consequences. Therefore, the prevalence of both symptoms and outcomes between children with anterior stroke (AS) and posterior stroke (PS) was also analyzed in the study. The comparison between children with posterior and patients with anterior stroke revealed that central type facial nerve palsy was a more common symptom of stroke in children with anterior stroke (59% vs. 27% in posterior stroke, *p* = 0.059). A significant difference was found for other symptoms of AIS, which were more frequent in children with posterior stoke compared to those with anterior stroke (91% vs. 17%, *p* < 0.001) (Table 5). Hemiplegia was observed only in children with anterior stroke. The analysis of the prevalence of stroke symptoms revealed no differences between girls and boys with AS (Table 5).

In the case of post-stroke outcomes, hemiparesis was observed in 71% children with anterior stroke compared to 46% of children with posterior stroke. Seizures after stroke concerned both posterior and anterior location with comparable frequency. Both aphasia and motor disturbances other than hemiparesis were found in 18% of children with posterior stroke, and in 9% of children with anterior stroke. The demonstrated differences were not significant. Similarly, the frequencies of post-stroke consequences did not significantly differ between girls and boys with anterior stroke.

## 4. Discussion

In our study, a slight predominance of males was observed (58% vs. 42% girls). Male sex was more prevalent in all age-subgroups of analyzed patients with AIS, with the greatest proportion of boys in the subgroup of infants–toddlers, and then the adolescents. In turn, the male proportion was extremely high in the case of the POCI and TACI stroke subgroups, while girls predominated in the LACI stroke subgroup. The impact of sex on stroke risk differs between studies—most authors found a statistically relevant rise in risk among boys [3,20,21,22]. Other studies which were conducted in Europe, the Americas and Asia also revealed that 56–60% of patients with AIS were males, similarly to our study [8,20,23,24,25]. A higher risk of head injury among boys cannot account fully for this disparity [3,22,26]. Many potential risk factors and mechanisms were stated as the cause of the sex differences, including hormonal, genetic and epigenetic ones. Single reports on the correlation of selected genetic polymorphisms with the neurological post-stroke consequences in children, including epilepsy or recurrent stroke, are available [27,28]. On the other hand, a similar crude incidence rate and age-standardized rate of AIS was found for boys as for girls in a prospective study performed in 96 children from southern England [5]. The authors demonstrated also that the age at AIS onset did not vary significantly by sex [5].

In the present study, the prevalence of stroke subtypes differentiated the girls with AIS from the boys with AIS. POCI stroke was significantly more common in boys. The male:female ratio in the POCI subgroup of pediatric patients with AIS was 10:1, while in the LACI subgroup it was 0.78:1. FCA, which is a risk factor specific to childhood stroke, was observed in 71% of analyzed male patients and in 60% of girls, but the difference was not significant. The mean age of stroke occurrence was comparable between the sexes. We found that the occurrence of specific symptoms at AIS onset did not differ between sexes in the whole group. The most frequent symptoms observed in both sex subgroups were as follows: hemiparesis (89% of girls vs. 79% of boys), disturbances of consciousness (70% of girls vs. 67% of boys), and VII nerve palsy (54% of girls vs. 56% of boys). Such a distribution of AIS symptoms is consistent with the stroke subtypes experienced by these children. We observed also that central type facial nerve palsy and other symptoms of AIS were more frequent in boys according to stroke subtypes (*p* = 0.050 and *p* < 0.001, respectively). The posterior circulation stroke in children is clinically characterized by nonspecific symptoms, e.g., ataxia, vertigo, vomiting and altered consciousness. In our study group, almost 70% of children with POCI stroke experienced disturbances of consciousness as a stroke symptom, of whom 85% were boys. Similarly, in the study by Carey et al. [26], 71% of children with posterior circulation stroke had impaired consciousness.

As for the AIS symptoms prevalence, the results from the present study were similar to those obtained in the Canadian pediatric population [20]. However, there are also some discrepancies in terms of the frequencies of first clinical presentations in the general pediatric stroke population between our study and other studies on pediatric stroke. It was previously described that seizures (occurring with the frequency from 52% to 58%), hemiplegia or hemiparesis, and headaches dominated as the presenting features at AIS onset, whereas cranial nerve palsy was present only in about 5% of cases [23,24]. The different ethnic backgrounds of the analyzed populations may be an explanation for this fact.

The literature’s data analyzing post-stroke consequences depending on sex in the group of pediatric patients are scarce. Most authors did not find any significant correlations [22,23,29]. In general, long-term neurologic consequences were observed in 31–70% of pediatric stroke patients [21,22,23,24,25,27,28,29,30]. The main goal of our study was to assess whether sex may be a predictor of a higher frequency of some post-stroke consequences, i.e., motor disability, seizures or aphasia, and the presented results show some tendencies that may be helpful in the future assessment of risk for post-stroke outcomes. Knowledge on post-stroke outcomes with regard to sex may lead to beneficial interventions to improve neurologic outcome after stroke. It is important to note that in adult patients with AIS, even after adjusting the analysis for other factors, women have worse outcomes than men [18,25,31,32]. A recent study presented by Geng et al. [32], including 228 young adult (18–50 years old) AIS patients from Eastern China, proved the female sex to be a risk factor for both death at the acute phase of the disease and dependency 12 months from the stroke onset.

In this retrospectively analyzed group of pediatric patients, similar numbers of girls and boys both in the whole group as well as in age subgroups had one or more post-stroke deficits. The most frequent AIS outcomes were persistent hemiparesis and post-stroke epilepsy. In the total group as well as in the age subgroups, no statistical differences were observed in the prevalence of these outcomes between the boys and girls. However, some tendencies were demonstrated, especially in the case of post-stroke seizures, which were present in 67% of girls and in 18% of boys in the group of infants and toddlers, but the difference was close to significance. Seizures after stroke appeared significantly more commonly in girls with TACI stroke, while only in one girl with LACI stroke and in the only girl with POCI stroke (*p* = 0.022). A study performed in Turkey also demonstrated that post-stroke epilepsy was more common in AIS girls, whereas no significant sex differences were observed between AIS children with epilepsy and AIS children without epilepsy [33]. In the study by Laugesaar et al. [34], cortical lesions, and involvement of the thalamus and temporal lobe, were reported to be independent predictors of post-stroke epilepsy in 73 children, of whom 53% were boys. Cortical involvement was also significantly related to nonmotor outcomes, including cognitive/behavioral outcomes, visual deficits and epilepsy, in a group of perinatal stroke from Canada [35].

In patients with clinical aphasia, the anterior system is primarily responsible for the clinical picture. In our study, aphasia was observed in two girls and in no boy in the infant–toddler group; both of the girls suffered from TACI stroke. However, in both the children and adolescents subgroups, aphasia was present only in boys. The Fisher’s exact test revealed that the difference was close to statistical significance when infant–toddlers and adolescents were analyzed. In turn, despite the fact that girls mature more rapidly in verbal abilities than boys, Ilves et al. [36] demonstrated no differences between sexes in language skills when comparing perinatal and the childhood strokes. On the other hand, Westmacott et al. [37], in a group of 26 children with neonatal AIS, observed the sex difference in long-term cognitive outcome. Boys showed significantly poorer overall intellectual ability, nonverbal reasoning, and processing speed compared to a matched group of girls. Interestingly, the authors found this difference only during the school-age assessment [37].

Recently, in a study by Chelse et al. [38], sex did not differentiate the subgroups of children with post-stroke headaches from those with headaches of another origin. In turn, in a Nigerian study based on children with sickle cell disease, the male sex was not correlated with subsequent stroke [39]. Arteriopathy is proven to be the most common identifiable cause of AIS (up to 60%). It is correlated with a higher risk of recurrence, and, as a result, leads to possible worse outcomes and an increase in death rate [1,20,40]. Our previous study regarding post-stroke epilepsy in children demonstrated that all cases with late remote seizures had FCA [11]. In the present study group, no recurrences were observed. This may be due to the fact that almost one fifth of the patients had heart diseases and were treated with antithrombotics for prophylaxis.

One of the most interesting findings of our study was the higher prevalence of posterior circulation stroke in boys compared with girls. Previously, Ganesan et al. [41] demonstrated that over 77% of children with posterior stroke were boys. These male patients had vertebrobasilar arterial abnormalities which are often multifocal. Over half of the analyzed group from the UK had no impairment at follow-up, whereas recurrent stroke occurred in 20% of patients [41]. On the other hand, in Mackay et al.’s study [42], the recurrence rate was significantly higher in children with posterior stroke (i.e., in 52%), of which 43% had posterior circulation stroke as the subsequent event, and in 57% of patients anterior circulation stroke recurred. In our study, neither posterior nor anterior stroke was associated with a higher frequency of any of the post-stroke outcome. Previously, some data indicated that injuries of posterior areas, particularly the cerebellum, may have an impact on language processing. Posterior strokes may result in cognitive deficits, including neurolinguistic components [43]. Surprisingly, in our study boys suffering from POCI stroke had no features of cerebellar syndrome during the follow-up. The latest data could bring some interesting and, more importantly, applicable conclusions for practitioners looking after stroke children both in the acute phase of the disease and then also in ambulatory care; e.g., more intensive and prolonged speech therapy should be planned for boys after stroke.

There are a few limitations of this study. Firstly, the number of pediatric patients with AIS was low, which forced the usage of non-parametric tests in the statistical analyses. The results obtained are supposed to be treated with caution. In addition, we attempted to obtain specific data from the medical history of patients with AIS, but the retrospective nature of the study may cause some information to be lacking. Moreover, differences in clinical outcome and presentation due to sex may be mediated by stroke distribution/territory. The analyzed group was additionally not homogenous with respect to age, which resulted mainly from the rarity of the disease. However, this is consistent with other studies describing AIS patients with ages ranging from early infancy to early adulthood. To overcome the problem of a wide age range, the recruited AIS group was divided into three age subgroups. Lastly, the length of follow-up period may also have impact on the results. Therefore, we evaluated just the existence (quality), not degree (quantity), of the selected consequences of AIS.

## 5. Conclusions

In conclusion, sex may play an important role in predicting the most likely type of stroke in pediatric patients. In the present study we hypothesized that sex may have an impact on the occurrence of specific post-stroke outcome. The analyses made did not reveal significant differences, or the results were close to the boundary of significance both in the total group and in the age subgroups. On the other hand, the prevalence of post-stroke seizures differed significantly between stroke subtypes in girls, whereas post-stroke hemiparesis significantly differentiated AIS subtypes in boys.

The knowledge concerning the possible impact of sex on post-stroke outcomes is noteworthy since it may contribute to more effective therapeutic actions during the acute phase of the disease and in the management of specific post-stroke outcomes. Many interfering factors which have an impact on stroke in adults do not concern children, therefore they cannot be extrapolated to pediatric patients. Further prospective, pediatric population-based studies on larger and more age-homogenous pediatric patient groups are needed to confirm these pilot results and to obtain new data explaining the impact of sex on AIS course, as well as on post-stroke period. Such studies may help in explaining some of the sex disparities, which were also demonstrated by other authors.

## Figures and Tables

**Table 1:** Proportions of male sex in total group and subgroups according to age and stroke subtype.

Group/Subgroup of AIS Patients	Number of Male Subjects	Male:Female Ratio	Proportion of Male Subjects	Lower and Upper Limits of 95%CI for Proportion of Male Subjects
Total (*N* = 89)	52	1.41:1	0.584	(0.475–0.686)
Infant–Toddlers (*N* = 17)	11	1.83:1	0.647	(0.386–0.847)
Children (*N* = 41)	22	1.16:1	0.537	(0.376–0.690)
Adolescents (*N* = 31)	19	1.58:1	0.613	(0.438–0.763)
LACI (*N* = 23)	10	0.78:1	0.435	(0.239–0.651)
TACI (*N* = 27)	18	2:1	0.667	(0.460–0.828)
PACI (*N* = 28)	14	1:1	0.500	(0.311–0.689)
POCI (*N* = 11)	10	10:1	0.909	(0.571–0.995)

AIS—arterial ischemic stroke; CI—confidence interval.

**Table 2:** Characteristics of entire analyzed group of children with AIS depending on gender.

	Total Group *N* = 89	Girls with AIS *N* = 37 (42%)	Boys with AIS *N* = 52 (58%)	*p*
Age at stroke onset (years), M ± SD	8.40 ± 5.56	8.40 ± 5.16	8.39 ± 5.89	0.997
Median, (Min.–Max.)	8 (0.11–18)	8 (1–17)	7 (0.11–18)	
Interquartile range (IQR)	11 (3–14)	9.25 (3–12.5)	11 (4–15)	
Birth weight (g), M ± SD	3093.39 ± 532	2956 ± 514	3186 ± 531	0.094
FCA, *n* (%)	59 (66)	22 (60)	37 (71)	0.265
Heart disease, *n* (%)	14 (16)	6 (16)	8 (15)	1.000
Number of infarct foci, *n* (%)				1.000
One	62 (70)	26 (70)	36 (69)	
Two or more	13 (14)	6 (16)	7 (14)	

AIS—arterial ischemic stroke; M—mean; SD—standard deviation; FCA—focal cerebral arteriopathy.

**Table 3:** Frequencies of AIS symptoms in girls and boys according to stroke subtypes.

**GIRLS**	**Total** ***N*** **= 37**	**LACI** ***N*** **= 13**	**TACI** ***N*** **= 9**	**PACI** ***N*** **= 14**	**POCI** ***N*** **= 1**	***p*** *
**SYMPTOMS OF AIS**						
Hemiplegia, *n* (%)	3 (8)	0 (0)	2 (22)	1 (7)	0 (0)	0.301
Hemiparesis, *n* (%)	33 (89)	12 (92)	7 (78)	13 (93)	1 (100)	0.582
Central type facial nerve palsy, *n* (%)	20 (54)	7 (54)	4 (44)	8 (57)	1 (100)	0.953
Consciousness disturbances, *n* (%)	26 (70)	9 (69)	8 (89)	8 (57)	1 (100)	0.389
Headache, vertigo, *n* (%)	10 (27)	3 (23)	1 (11)	5 (36)	1 (100)	0.232
Aphasia, *n* (%)	14 (38)	6 (46)	4 (33)	4 (29)	1 (100)	0.499
Convulsions, *n* (%)	6 (16)	1 (8)	2 (22)	2 (14)	1 (100)	0.239
Other symptoms, *n* (%)	6 (16)	3 (23)	1 (11)	1 (7)	1 (100)	0.185
**BOYS**	**Total** ***N* = 52**	**LACI** ***N* = 10**	**TACI** ***N* = 18**	**PACI** ***N* = 14**	**POCI** ***N* = 10**	*p* *
**SYMPTOMS OF AIS**						
Hemiplegia, *n* (%)	6 (12)	1 (10)	3 (17)	2 (14)	0 (0)	0.781
Hemiparesis, *n* (%)	41 (79)	9 (90)	15 (83)	10 (71)	7 (70)	0.652
Central type facial nerve palsy, *n* (%)	29 (56)	5 (50)	13 (72)	9 (64)	2 (20)	**0.050**
Consciousness disturbances, *n* (%)	35 (67)	6 (60)	14 (78)	9 (64)	6 (60)	0.680
Headache, vertigo, *n* (%)	18 (35)	4 (40)	8 (44)	2 (14)	4 (40)	0.322
Aphasia, *n* (%)	22 (42)	3 (30)	9 (50)	6 (43)	4 (40)	0.794
Convulsions, *n* (%)	9 (17)	1 (10)	3 (17)	3 (17)	2 (20)	0.959
Other symptoms, *n* (%)	17 (33)	1 (10)	3 (17)	4 (29)	9 (90)	**<0.001**

AIS—arterial ischemic stroke; LACI—lacunar anterior circulation infarct; TACI—total anterior circulation infarct; PACI—partial anterior circulation infarct; POCI—posterior circulation infarct; *—difference in frequency of particular AIS symptom between stroke subtypes. Significant differences are in bold.

**Table 4:** Frequencies of post-stroke outcomes in girls and boys regarding stroke subtypes.

**GIRLS**	**Total** ***N*** **= 37**	**LACI** ***N*** **= 13**	**TACI** ***N*** **= 9**	**PACI** ***N*** **= 14**	**POCI** ***N*** **= 1**	***p*** *
**POST-STROKE OUTCOMES**						
Hemiparesis, *n* (%)	27 (73)	7 (54%)	9 (100)	10 (71)	1 (100)	0.089
Seizures, *n* (%)	10 (27)	1 (8)	5 (56)	3 (23)	1 (100)	**0.022**
Aphasia, *n* (%)	2 (5)	0 (0)	2 (22)	0 (0)	0 (0)	0.108
Other motor disturbances, *n* (%)	4 (11)	1 (8)	1 (11)	1 (7)	1 (100)	0.170
Number of post-stroke outcomes, *n* (%)						0.160
None	8 (22)	5 (38)	0 (0)	3 (21)	0 (0)	
One or more	29 (78)	8 (62)	9 (100)	11 (79)	1 (100)	
**BOYS**	**Total** ***N* = 52**	**LACI** ***N* = 10**	**TACI** ***N* = 18**	**PACI** ***N* = 14**	**POCI** ***N* = 10**	*p* *
**POST-STROKE OUTCOMES**						
Hemiparesis, *n* (%)	33 (64)	4 (40)	16 (89)	9 (64)	4 (40)	**0.026**
Seizures, *n* (%)	10 (19)	1 (10)	5 (28)	2 (14)	2 (20)	0.721
Aphasia, *n* (%)	7 (14)	0 (0)	3 (17)	2 (14)	2 (20)	0.668
Other motor disturbances, *n* (%)	5 (10)	0 (0)	2 (11)	2 (14)	1 (10)	0.855
Number of post-stroke outcomes, *n* (%)						0.074
None	13 (25)	5 (50)	2 (11)	2 (14)	4 (40)	
One or more	39 (75)	5 (50)	16 (89)	12 (86)	6 (60)	

AIS—arterial ischemic stroke; LACI—lacunar anterior circulation infarct; TACI—total anterior circulation infarct; PACI—partial anterior circulation infarct; POCI—posterior circulation infarct; *—difference in the prevalence of particular outcome between stroke subtypes. Significant differences are in bold.

**Table 5:** Frequencies of AIS symptoms, depending on AIS location (posterior vs. anterior).

SYMPTOMS OF AIS	Children with PS *N* = 11	Children with AS *N* = 78	*p* *	Girls with AS *N* = 36	Boys with AS *N* = 42	*p* **
Hemiplegia, *n* (%)	0 (0)	9 (12)	0.594	3 (8)	6 (14)	0.494
Hemiparesis, *n* (%)	8 (73)	66 (85)	0.387	32 (89)	34 (81)	0.367
Central type facial nerve palsy, *n* (%)	3 (27)	46 (59)	**0.059**	19 (53)	27 (64)	0.360
Consciousness disturbances, *n* (%)	7 (64)	54 (69)	0.736	25 (69)	29 (69)	1.000
Headache, vertigo, *n* (%)	5 (45)	23 (29)	0.284	9 (25)	14 (33)	0.464
Aphasia, *n* (%)	5 (45)	31 (40)	0.522	13 (36)	18 (43)	0.644
Convulsions, *n* (%)	3 (27)	12 (15)	0.387	5 (14)	7 (17)	0.765
Other symptoms, *n* (%)	10 (91)	13 (17)	**<0.001**	5 (14)	8 (19)	0.762

AS—anterior stroke; PS—posterior stroke; *—children with PS vs. children with AS; **—girls with AS vs. boys with AS. Analysis was performed only between sex subgroups with AS since only one girl had posterior stroke. Significant differences are in bold.
